# Chromosome genomics facilitates the marker development and selection of wheat-*Aegilops biuncialis* addition, substitution and translocation lines

**DOI:** 10.1038/s41598-023-47845-8

**Published:** 2023-11-22

**Authors:** András Farkas, Eszter Gaál, László Ivanizs, Nicolas Blavet, Mahmoud Said, Kateřina Holušová, Kitti Szőke-Pázsi, Tamás Spitkó, Péter Mikó, Edina Türkösi, Klaudia Kruppa, Péter Kovács, Éva Darkó, Éva Szakács, Jan Bartoš, Jaroslav Doležel, István Molnár

**Affiliations:** 1 Department of Biological Resources, Centre for Agricultural Research, Eötvös Lóránd Research Network, Martonvásár, 2462 Hungary; 2 Institute for Experimental Botany of the Czech Academy of Sciences, Centre of Plant Structural and Functional Genomics, 779 00 Olomouc, Czech Republic; 3 Field Crops Research Institute, Agricultural Research Centre, 9 Gamma Street, Giza, Cairo 12619 Egypt

**Keywords:** Plant breeding, Plant molecular biology, Plant breeding, Plant genetics

## Abstract

The annual goatgrass, *Aegilops biuncialis* is a rich source of genes with considerable agronomic value. This genetic potential can be exploited for wheat improvement through interspecific hybridization to increase stress resistance, grain quality and adaptability. However, the low throughput of cytogenetic selection hampers the development of alien introgressions. Using the sequence of flow-sorted chromosomes of diploid progenitors, the present study enabled the development of chromosome-specific markers. In total, 482 PCR markers were validated on wheat (Mv9kr1) and *Ae. biuncialis* (MvGB642) crossing partners, and 126 on wheat-*Aegilops* additions. Thirty-two markers specific for U- or M-chromosomes were used in combination with GISH and FISH for the screening of 44 Mv9kr1 × *Ae. biuncialis* BC_3_F_3_ genotypes. The predominance of chromosomes 4M and 5M, as well as the presence of chromosomal aberrations, may indicate that these chromosomes have a gametocidal effect. A new wheat-*Ae. biuncialis* disomic 4U addition, 4M(4D) and 5M(5D) substitutions, as well as several introgression lines were selected. Spike morphology and fertility indicated that the *Aegilops* 4M or 5M compensated well for the loss of 4D and 5D, respectively. The new cytogenetic stocks represent valuable genetic resources for the introgression of key genes alleles into wheat.

## Introduction

Bread wheat (*Triticum aestivum* L; 2*n* = 6*x* = 42; AABBDD) is the third most important crop after rice and maize and is essential in the human diet. Thousands of years of cultivation and modern breeding in the twentieth century resulted in improved yield and quality^1^. However, it reduced the available genetic diversity within elite germplasm, making it difficult to identify the best allele combinations to produce superior cultivars.

Goatgrasses (*Aegilops*) are close relatives of *Triticum,* making them promising gene sources for increasing wheat genetic diversity through interspecific hybridization^2^. In the 23 species of the genus, seven different genomes (C, D, M, N, S, T, U) were identified, and 12 of them contain U and/or M genomes^3^. The allotetraploid *Ae. biuncialis* Vis. (2*n* = 4*x* = 28, U^b^U^b^M^b^M^b^) has a high ability for ecological adaptation^3–5^. Together with its diploid progenitors *Ae. comosa* Sm. in Sibth. & Sm. (2*n* = 2*x* = 14; MM) and *Ae. umbellulata* Zhuk. (2*n* = 2*x* = 14; UU), they are attractive sources of agronomically important genes. *Ae. biuncialis* has been reported as gene source to improve wheat drought and salt tolerance^6–8^, as well as resistance to pests and disseases such as barley yellow dwarf luteovirus^9^, powdery mildew^10^, yellow rust^11^, brown rust^12^, leaf rust^13^, and stem rust^14^. Numerous data are also available for improving grain quality traits such as dietary fibre, β-glucan, protein content, Zeleny sedimentation value, wet gluten content, grain hardness, and micronutrient content^15–17^. Despite the high genetic potential, only a few wheat-*Ae. biuncialis* hybrids and addition lines have been developed^18–20^. Recently, a set of winter wheat (Mv9kr1)-*Ae. biuncialis* addition lines carrying chromosomes 1U^b^, 2U^b^, 3U^b^, 2M^b^, 3M^b^ and 7M^b^^18,21,22^ has been developed. Tan et al.^23^ produced a wheat (‘Chuannong 19’)-*Ae. biuncialis* partial amphiploid and used it to create addition lines representing chromosomes 6U^b^^24^, 1U^b^^15^ and 2M^b^^10^, while Song et al.^25^ developed a disomic addition line 5M^b^. The production of wheat-*Ae. biuncialis* substitution and translocation lines has been reported even less frequently. Farkas et al.^16^ produced a wheat-*Ae. biuncialis* 3M^b^(4B) substitution line and a 3M^b^·4BS translocation line. It is estimated that only 4–5 *Ae. biuncialis* accessions are represented in the previously published wheat-*Ae. biuncialis* introgression lines. However, recent two diversity analyses of *Ae. biuncialis* accessions from different ecogeographical habitats revealed that this species has a high genetic variation for heading date, seed quality traits, and stem rust resistance available for the introgression breeding programs^4,5,14^.

The ability to screen large populations for the presence of introgressed chromatin is essential for producing wheat-alien translocations. Molecular cytogenetic methods such as genomic in situ hybridization (GISH) and fluorescence in situ hybridization (FISH) are probably the most popular approaches for characterizing alien chromatin in the wheat genetic background. Thus, GISH and FISH have been widely used to identify alien introgressions, including wheat-*Ae. biuncialis* addition and translocation lines^10,16,22,26^. Although molecular cytogenetic methods cannot be excluded from the selection process, their disadvantages, such as time and labor intensity, make screening large populations difficult. Furthermore, due to the low resolution of FISH and GISH, small alien chromosome segments (micro-introgressions) responsible for the trait of interest are undetectable ^27^.

Selection assisted by molecular markers would facilitate the much higher throughput identification of M- and U-genome chromatin. Earlier, testing wheat Amplified Fragment Length Polymorphism (AFLP), Restriction Fragment Length Polymorphism (RFLP), and Simple Sequence Repeat (SSR) molecular markers on *Aegilops* species was a suitable approach to detect introgressed chromatin^28,29^. Sequence-Specific Amplified Polymorphism (S-SAP) markers, which take advantage of the diversity of the integration sites of specific retroelements, provided an additional opportunity to identify transferred *Aegilops* chromosomes in wheat^30^. The use of gene-based markers can be even more effective, as demonstrated by Howard et al.^31^, who used wheat Conserved Ortholog Set (COS) markers to map QTLs determining the B-type starch granules content in *Ae. peregrina*. The markers specific for conserved orthologous genes facilitated gene introgression from U and M genome *Aegilops* species into wheat as well as the investigation of wheat-*Aegilops* macrosynteny at chromosomal level^32,33^. Molnár et al.^32^ used wheat-*Aegilops* chromosome addition lines to map a series of COS markers to the U and M genomes, demonstrating the utility of COS markers. Because the markers have known positions on the deletion bin map of wheat genomes, they allowed researchers to study wheat-*Aegilops* homeologous relationships^34^. In the last decade, PCR-based Landmark Unique Gene (PLUG) markers, another gene specific marker family, have become increasingly popular for detecting *Aegilops* chromatin in the wheat^15,33,35^. Until now, the majority of *Ae. biuncialis* markers tested were also wheat-specific, limiting their effectiveness. The development of *Aegilops*-specific markers would greatly aid in the detection of introgressed *Aegilops* chromatin in wheat.

Recent advances in flow cytometric sorting of plant mitotic chromosomes linked to next generation sequencing technologies^36^ provide an efficient strategy for producing chromosome-specific genomic resources in the case of large *Triticeae* genomes of agronomically important species such as wheat, barley and rye^37–39^.

Chromosome sorting and sequencing from wild species have also been reported^40,41^, including *Aegilops* species with U and M genomes^34,42–44^. Tiwari et al.^45^ flow sorted chromosome 5M^g^ from a wheat-*Ae. geniculata* disomic substitution line and sequenced it with Illumina. Using sequence assembly, the authors were able to annotate 4236 genes and develop 4538 SNPs on chromosome 5M^g^. Bivariate flow cytometry has also been used to dissect the genomes of the diploid progenitors *Ae. umbellulata* (U) and *Ae. comosa* (M) into individual chromosomes^34^. Draft sequence assemblies of the U and M chromosomes allowed the authors to develop molecular markers for specific cytogenetic positions of orthologous genes previously mapped by single gene FISH on the chromosomes^43^. Although these markers have been PCR validated on wheat-*Aegilops* addition lines, they have not been tested on prebreeding populations to select wheat-*Ae. biuncialis* introgression lines.

Motivated by the need to develop wheat-*Ae. biuncialis* introgression lines, our study aims to develop gene specific markers for *Aegilops* chromosomes and use them to screen a winter wheat BC_3_F_3_ pre-breeding population for the presence of alien chromatin.

With *Ae. umbellulata* chromosome-specific draft assemblies, three marker design strategies were used: (1) a set of deletion-bin mapped wheat Expressed Sequence Tag (EST) sequences was used to design markers specific for InDel regions polymorphic between wheat and *Aegilops* (InDEL markers). (2) The annotated gene models of *Ae. umbellulata* were also used for sequence similarity searches against wheat in order to develop Intron Targeting (IT) markers. (3) We also tested previously developed markers for specific cytogenetic positions on U- and M-genome chromosomes using single-gene FISH. Finally, a combination of PCR markers validated on wheat-*Aegilops* chromosome addition lines and molecular cytogenetic methods were used to detect alien chromatin in a winter wheat (Mv9kr1)-*Ae. biuncialis* MvGB642 BC_3_ population to identify and characterize new addition, substitution, and translocation lines.

## Results

### Development of PCR markers

**Figure 1:**
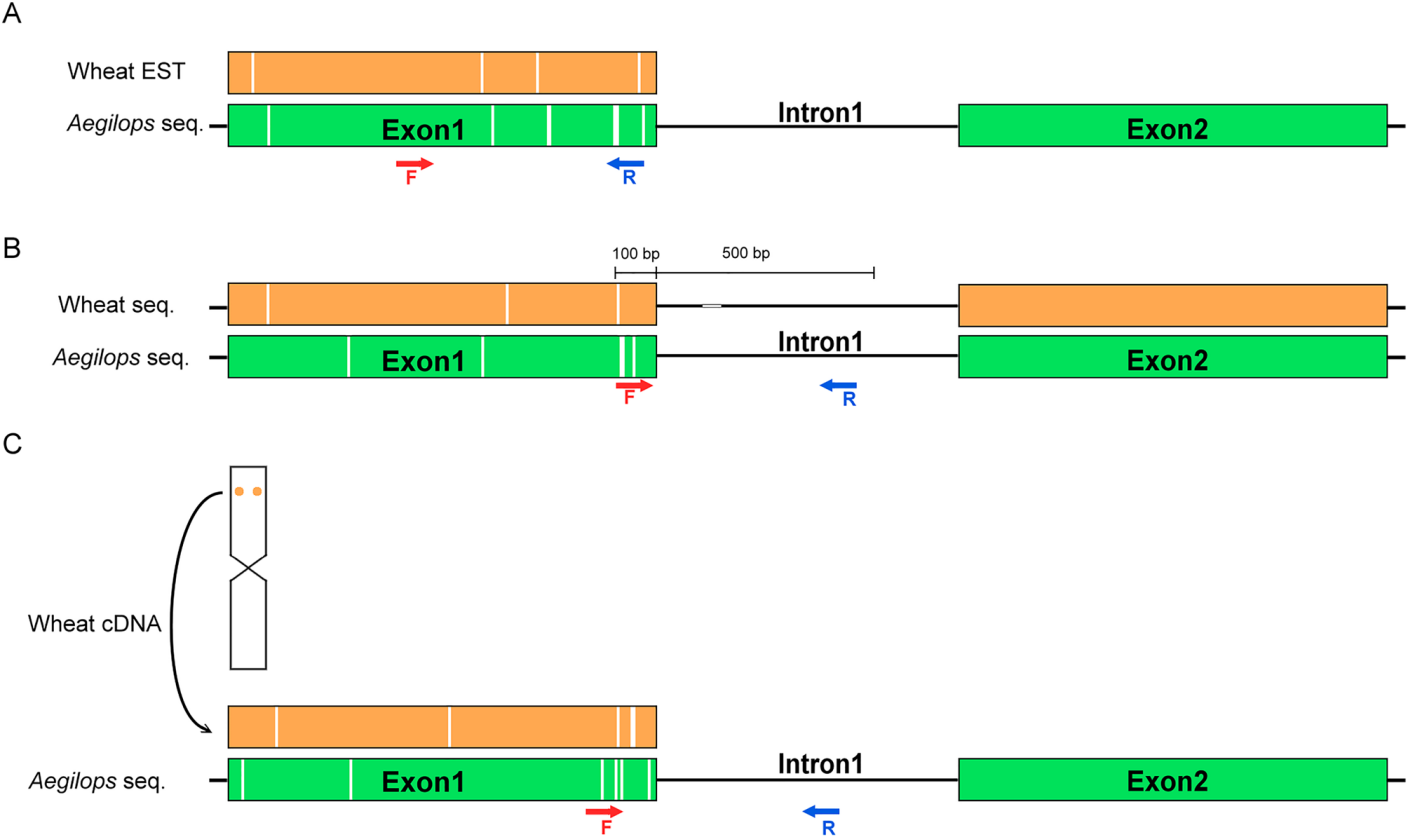
Development of gene-based molecular markers using *Ae. umbellulata* chromosome sequences. (**A**) EST-based markers: wheat EST sequences were aligned to *Ae. umbellulata* chromosome sequences and primers were designed for the polymorphic exon regions. (**B**) Intron-targeting (IT) markers: annotated gene models of *Ae. umbellulata* were mapped to hexaploid wheat pseudomolecules. Primer pairs were designed for the last 100 bp of the first exon and the first 500 bp of the first intron. (**C**) cDNA-based markers: the physically mapped cDNA sequences of wheat orthologous genes ^43^ were aligned to the *Ae. umbellulata* and *Ae. comosa* chromosome sequences, and PCR primers were designed for the polymorphic (> 2 bp InDel) exon and intron regions. *Aegilops* chromosome sequences are highlighted in green, wheat sequences in ocher yellow, and polymorphic regions in white. *F* forward primer, *R* reverse primer.

Sequence assemblies of *Ae. umbellulata* chromosomes enabled the development of new gene-specific markers that can support the *Ae. biuncialis* gene introgression into wheat. In this study, we used molecular markers developed by three different strategies (Fig. 1A–C).

The first strategy was based on the polymorphism between physically mapped wheat EST sequences and *Ae. umbellulata* chromosome sequences of (Fig. 1A). To generate EST markers, we aligned wheat EST sequences covering the wheat group I-VII chromosomes (sum of aligned ESTs = 4514; mean number of ESTs/chromosome: 644.8) to *Ae. umbellulata* chromosome contigs of Data S1*.* Using pairwise alignment, we identified polymorphic (> 5 bp InDels) hits with 338 ESTs on *Ae. umbellulata* chromosomes (1U:34, 2U:73, 3U:40, 4U:44, 5U:32, 6U:68, 7U:47). A total of 124 primer pairs out of the 313 primer pairs designed for the polymorphic regions of 204 ESTs were tested by PCR on the wheat (Mv9kr1), *Ae. biuncialis* (MvGB642), and *Ae. umbellulata* (AE740/03) (Fig. S1A, Table 1). Fifty markers (40.3%) produced polymorphic amplicons between wheat and *Aegilops* accessions (Data S2). In order to determine the chromosomal localization of the markers, we assigned the polymorphic markers to *Aegilops* chromosomes using wheat-*Aegilops* chromosome addition lines representing chromosomes 1U–7U and 1M–7M (Fig. S1B). Thirty-nine polymorphic markers were assigned to *Aegilops* chromosomes (Data S2.). Twenty-three markers were single-chromosome specific (1U: 9, 2U:1, 3U: 2, 4U:4, 5U:6, 6U:5, 7U: 7, 1M: 1, 5M: 2, 7M: 2), while three markers were detected on both the U and M genomes (1U/1M: BE497808, 7U/7M: BF483072, BE423703). These markers are considered suitable for selecting wheat lines with the corresponding *Aegilops* chromosomes during pre-breeding programs. (Table 1, Data S2).

**Table 1:** The summary of marker development.

		EST	IT	cDNA*	Total
In silico designed markers		313	15,475	274	16,062
PCR validated on crossing partners					
Tested		124	84	274	482
Polimorphic (+ /−)		22	21	52	95
Polimorphic (size)		28	8	84	120
PCR validated on additions					
Assigned		39	20	39	98
Markers/chr	1U	10	2	5	17
	2U	1	2	1	4
	3U	2	3	10	15
	4U	4	4	2	10
	5U	7	3	4	14
	6U	4	2	6	12
	7U	8	3	3	14
	1M	2	1	–	3
	2M	–	2	1	3
	3M	–	–	5	5
	4M	–	1	3	4
	5M	2	–	1	3
	6M	–	–	3	3
	7M	2	1	2	5
No. of markers used for MAS		3	10	19	32

The number of gene-based (EST, IT, and cDNA) markers developed in this study, PCR validated on crossing partner wheat (Mv9kr1) and *Ae. biuncialis* (MvGB642) genotypes, on wheat-*Aegilops* addition lines, and used in marker-assisted selection (MAS) systems.

*Markers selected from previous study by Said et al. ^43^.

In order to develop *Aegilops* chromosome-specific, Intron-targeting (IT) markers (Fig. 1B), the annotated gene models of *Ae. umbellulata* were mapped to the hexaploid wheat reference sequence (RefSeq v1.0^37^). When suitable (> 10 bp) wheat/*Aegilops* polymorphism was found in the first exon and intron regions, a primer pair was designed within the last 100 bp of the exon (forward primer) and the first 500 bp of the intron (reverse primer). Finally, 15,475 primer pairs (5 primer pairs/contig) were designed using 3095 *Ae. umbellulata* contigs (Table 1, Data S3).

Eighty-four randomly selected primer pairs (12 markers per chromosome) were tested by PCR on the parental genotypes (Mv9kr1 and *Ae. biuncialis* MvGB642), and 29 (34.5%) of them showed presence/absence (21) or length (8) polymorphism between *Aegilops* and wheat (Data S2). Twenty polymorphic markers were located on *Aegilops* chromosomes using wheat Chinese Spring (CS)-*Ae. umbellulata* 1U-7U and CS-*Ae. geniculata* 1M–7M addition lines (1U: 2, 2U: 3, 3U: 2, 4U: 4, 5U: 3, 6U: 2, 7U: 3, 1M: 1, 2M: 2, 4M: 1, 7M: 1) (Table 1); sixteen were single-chromosome specific, three were located on both the U- and M-genome chromosomes (2U/2M: *Ae2U14986.1*, 7U/7M: *Ae7U16619.2*, 5U/4M: *Ae5U23507.1*), and the marker *Ae4U242236.1* was detected on both the 4U and 6U chromosomes (Data S2).

The third type of markers was developed by Said et al.^43^ using wheat cDNAs that were single-gene FISH mapped to the U and M chromosomes of *Ae. umbellulata* and *Ae. comosa*, respectively. In this study, 19 markers were selected to characterize the Mv9kr1 × *Ae. biuncialis* MvGB642 BC_3_F_3_ generation (Data S2, Table 1).

In total, 16,062 primer pairs were designed using the three marker development strategies. 215 of the 482 markers tested on wheat and *Aegilops* parents showed polymorphisms. The chromosomal localization of 98 markers was determined using wheat-*Aegilops* addition lines. In order to trace the *Aegilops* chromosomes in the wheat background, 32 of these markers (3 EST, 10 IT, and 19 cDNA-based markers) were used to detect U or M chromosomes in the wheat-*Ae. biuncialis* BC_3_F_3_ population.

### Marker-assisted selection of wheat-*Ae. biuncialis* introgression lines

**Figure 2:**
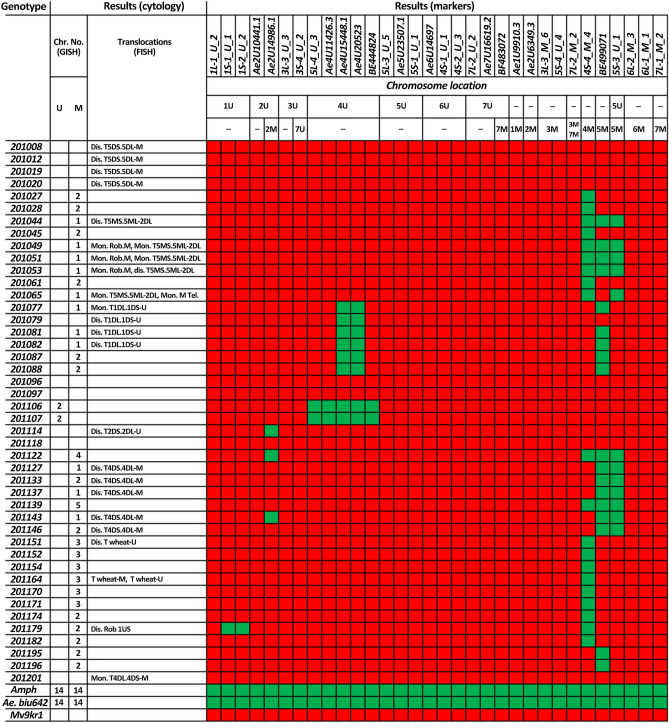
Chromosomal constitution of wheat-*Aegilops* BC_3_F_3_ genotypes, as well as the amphiploid (Amph) and their parental wheat (Mv9kr1) and *Ae. biuncialis* MvGB642 (Ae.biu642) genotypes determined using GISH, FISH and molecular markers. Abbreviations for GISH and FISH results: *Mon* monosomic, *Dis* disomic, *T* translocation, *Rob* Robertsonian translocation (centric fusion), *wheat* unidentified wheat chromosome. The green and red colors represent the presence or absence of the *Aegilops* chromosome specific PCR amplicon, respectively.

For tracking the individual *Aegilops* chromosomes in the wheat background, we chose markers that produced amplicons only on *Aegilops* chromosomes and tested them in 44 wheat-*Ae. biuncialis* BC_3_F_3_ hybrid progenies originated from crosses of hexaploid winter wheat line Mv9kr1 and *Ae. biuncialis* accession MvGB642 (Fig. S2). In order to validate the results of the 32 markers, sequential mcGISH and FISH were also performed on the BC_3_F_3_ population (results summarized in Fig. 2.).

Based on the markers, five of the 14 *Aegilops* chromosomes were detected in the pre-breeding population (Fig. 2; Data S4). Chromosomes 4M and 5M were most frequent in the population with 20 and 19 lines, respectively, containing these chromosomes. In contrast, the frequency of U-genome chromosomes was lower, with chromosomes 1U, 2U and 4U present in 1, 3 and 8 lines, respectively.

**Figure 3:**
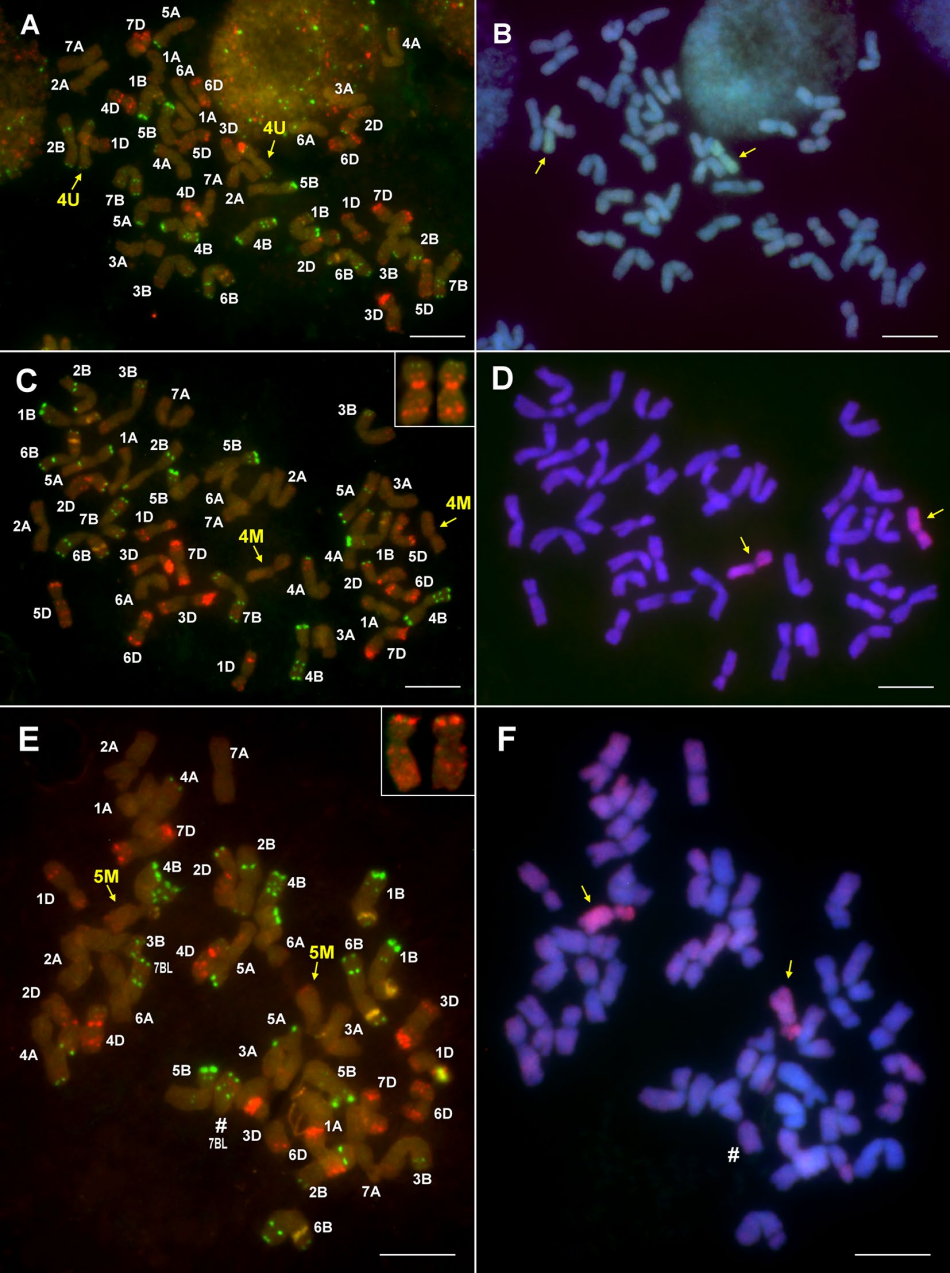
Molecular cytogenetic identification of Mv9kr1-*Ae. biuncialis* MvGB642 4U addition (**A,B**), 4M(4D) substitution (**C,D**), and 5M(5D) substitution (**E,F**) in the BC_3_F_3_ lines 201107, 201028 and 201195, respectively. FISH (**A,C,E**) was performed with Afa-family (red), pSc119.2 (green), and pTa71 (yellow) DNA repeat probes. The FISH pattern of the missing 4D (**C**) and 5D (**E**) chromosomes is shown in the inset. In the case of GISH (**B,D,F**), U- and M-genomic DNA probes were visualized with green and red fluorescence, respectively, while DAPI-stained wheat chromosomes were visualized in blue. Scale bar = 10 μm.

**Figure 4:**
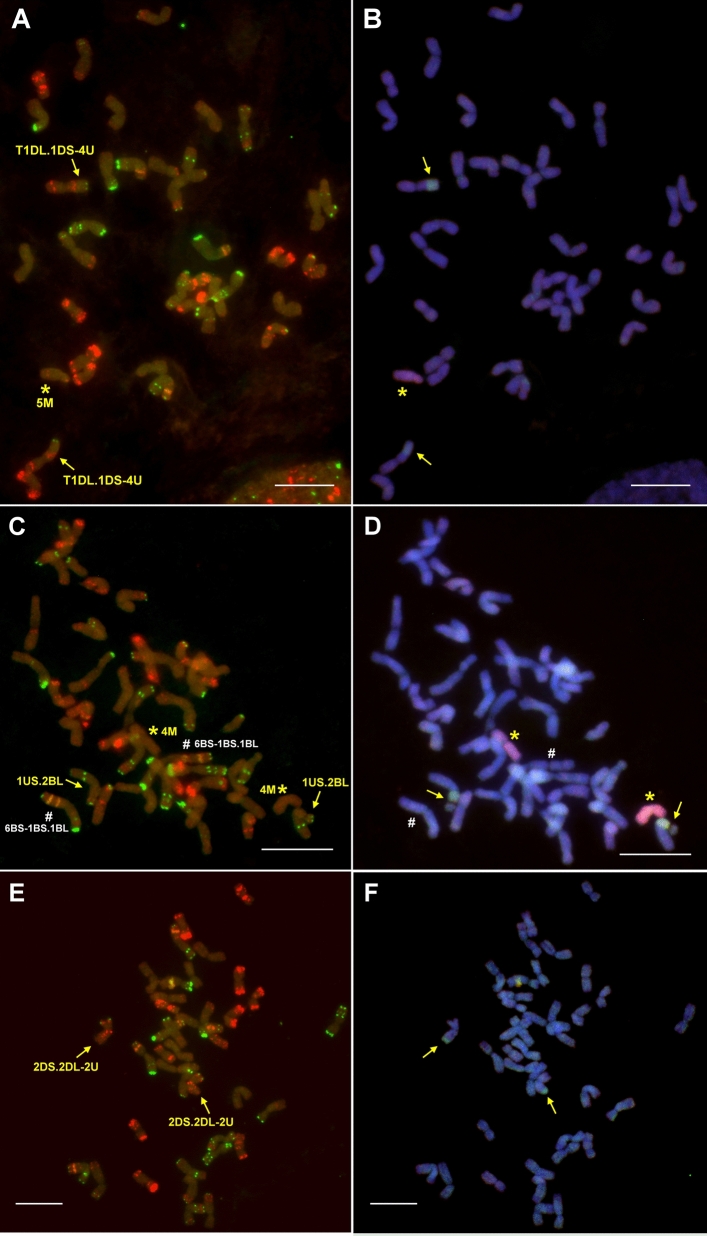
Identification of Mv9kr1-*Ae. biuncialis* MvGB642 1DL**·**1DS-4U (**A,B**), 1US**·**2BL (**C,D**), and 2DS**·**2DL-U (**E,F**) disomic translocations in the 201081, 201179, and 201114 BC_3_F_3_ lines, respectively. FISH (**A,C,E**) was performed using Afa-family (red), pSc119.2 (green), and pTa71 (yellow) repetitive DNA probes. On the GISH images (**B,D,F**), U- and M-chromosome fragments were visualized with green and red fluorescence, respectively, while DAPI-stained wheat chromosomes were visualized with blue. The presence of a wheat 1BL**·**1BS-6BS translocation (#) was detected (**C,D**). Scale bar = 10 μm.

Regarding the lines with 4U chromatin, two genotypes (201106 and 201107) contained more 4U chromatin than the remaining six as they produced PCR amplicons with all of the five 4U-specific markers. The presence of a pair of 4U chromosomes in these lines was confirmed by molecular cytogenetic analysis (the chromosomes were metacentric with strong telomeric pSc119.2 signals on both arms). Becase the lines contained 42 wheat and two U chromosomes, they were identified as wheat-*Ae. biuncialis* 4U disomic addition lines (Fig. 3A, B). The remaining six lines (201077, 201079, 201081, 201082, 201087, and 201088) produced amplicons by only two 4U-specific markers, *Ae4U15448.1* and *Ae4U20523,* indicating the presence of a 4U fragment (Fig. 2). Based on the specific Afa-family signals of normal wheat chromosome 1D (strong signals on subtelomeric and telomeric regions on the long and short arms, respectively), which are absent in these lines, the wheat-*Aegilops* translocation was identified as 1DL·1DS-4U translocation (Fig. 4A, B). In additio to the 1DL·1DS-4U translocation, *Aegilops* chromosome 5M was detected in five of these lines, while line 201079 contained the translocation in disomic form with no additional *Aegilops* chromosomes.

The line 201179 was identified as a 1US·2BL disomic centric fusion by the presence of a satellite, a faint pTa71 signal on the U chromosome arm typical for 1US, and the pSc119.2 FISH pattern on the wheat chromosome arm. The line also carried two 4M chromosomes (Fig. 4C, D). The line 201114 carried a pair of 2DS·2DL-U translocation. As the 2U-specific marker *Ae2U14986.1* (Fig. 3) produced amplicons in this line, the translocation was identified as a 2DS·2DL-2U (Fig. 4E, F).

The application of GISH in combination with the marker specific for chromosome 4M (*4S-4_M_4*) indicated that six of the 20 lines 4M (201027, 201028, 201045, 201061, 201174, and 201182) contained the *Aegilops* chromosome in disomic form. One of the six lines (201027) had 42 chromosomes. After rehybridization with DNA repeat probes (pSc119.2, Afa-family, pTa71), the presence of the 4M chromosome in disomic form was confirmed, as was the absence of wheat chromosome 4D, which has a typical hybridization pattern with pSc119.2 and Afa family repeats (Fig. 3C, D). This genotype was thus identified as a 4M(4D) disomic substitution line.

Using the 5M chromosome-specific marker *BE499071*, two genotypes (201195, 201196) were selected that contained two 5M but also had 42 chromosomes according to GISH. FISH confirmed the presence of disomic chromosome 5M (pTa71 signal in the telomeric region on the short arm and a relatively weak Afa-family signal in the telomeric and subtelomeric regions on the long arm) and the absence of chromosome 5D in these lines. These lines were thus identified as 5M(5D) disomic substitution lines (Fig. 3E, F). It should also be noted that instead of chromosome 7B, only one 7BL telosome was present in these lines.

**Figure 5:**
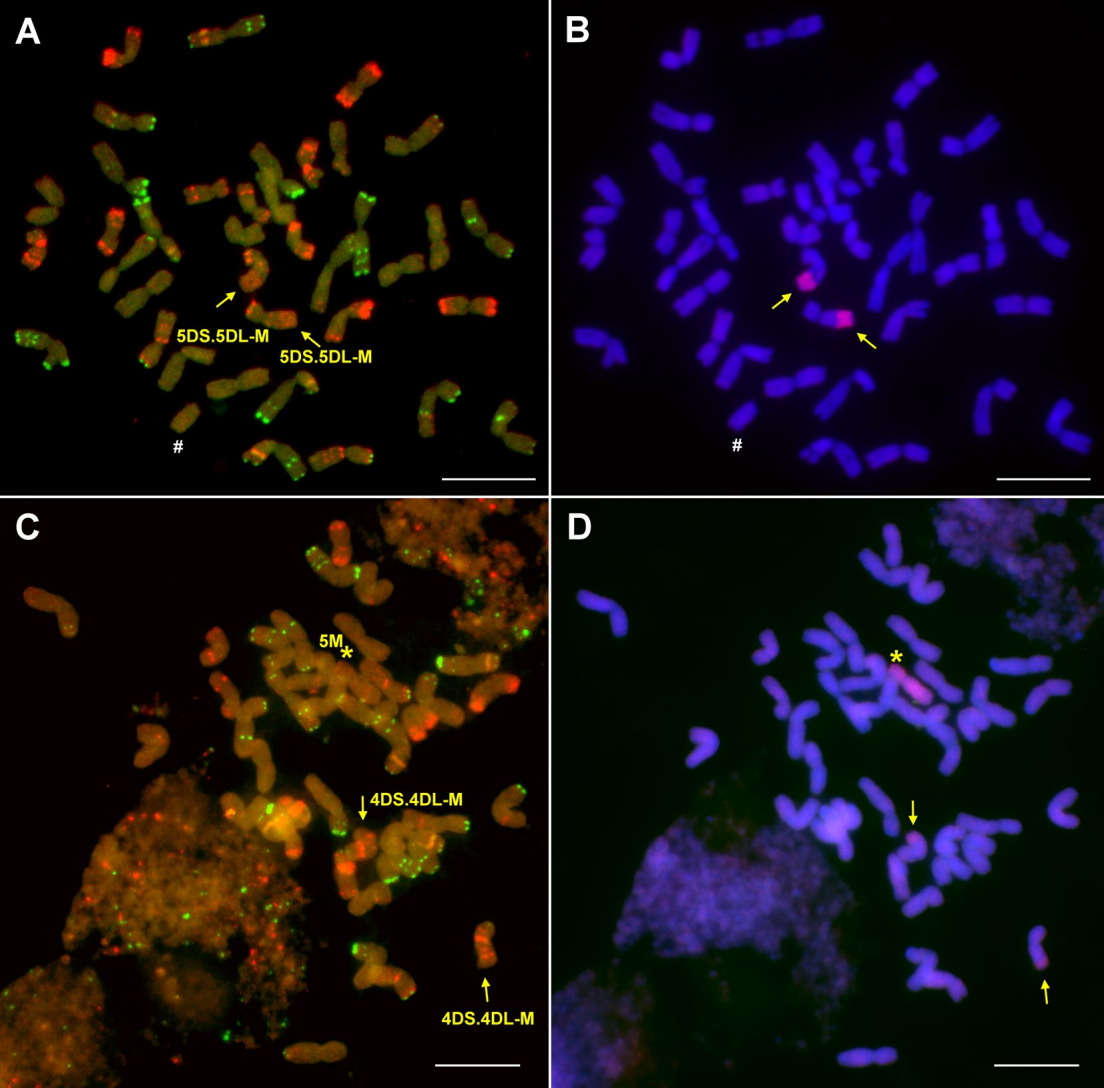
Molecular cytogenetic identification of Mv9kr1-*Ae. biuncialis* MvGB642 disomic 5DS·5DL-M (**A,B**) and 4DS·4DL-M translocations (**C,D**) in the 201020 and 201137 BC_3_F_3_ lines, respectively. FISH (**A,C**) was performed with Afa-family (red), pSc119.2 (green), and pTa71 (yellow) repetitive DNA probes. On the GISH images (**B,D**), M-chromosome fragments were visualized with red fluorescence, while DAPI-stained wheat chromosomes were visualized with blue. Asterisk: chromosome 5M. Scale bar = 10 μm.

GISH analysis revealed that a short M-genome segment had recombined with the terminal part of a wheat chromosome in four plants (201008, 201012, 201019 and 201020) (Figs. 2, 5A). The wheat segment showed strong telomeric Afa-family signals in the short arm, pericentromeric, weak interstitial and subtelomeric signals in the long arm, and very weak Afa-family signals in the the *Aegilops* segment (Fig. 5B), indicating that these lines carry a disomic 5DS·5DL-M translocation.

GISH and FISH also detected a 5MS·5ML-D translocation in five lines, two of which (201044, 201053) contained the translocation in disomic form (Fig. S3 A-B). Three of the five lines (201049, 201051, 201053) with the 5MS·5ML-D translocation also carried an unidentified centric fusion between a wheat and an M-genome chromosome arm (Fig. S3A, B). These lines also contained a 4M chromosome.

Finally, a disomic 4DS·4DL-M translocation was also identified by GISH and FISH in five BC_3_F_3_ plants (not supported by markers), which also contained an extra chromosome 5M in disomic (201133, 201137, 201146) or monosomic (201127, 201143) form (Figs. 3, 5C, D).

## Morphological traits of wheat-*Ae. biuncialis* chromosome lines

**Figure 6:**
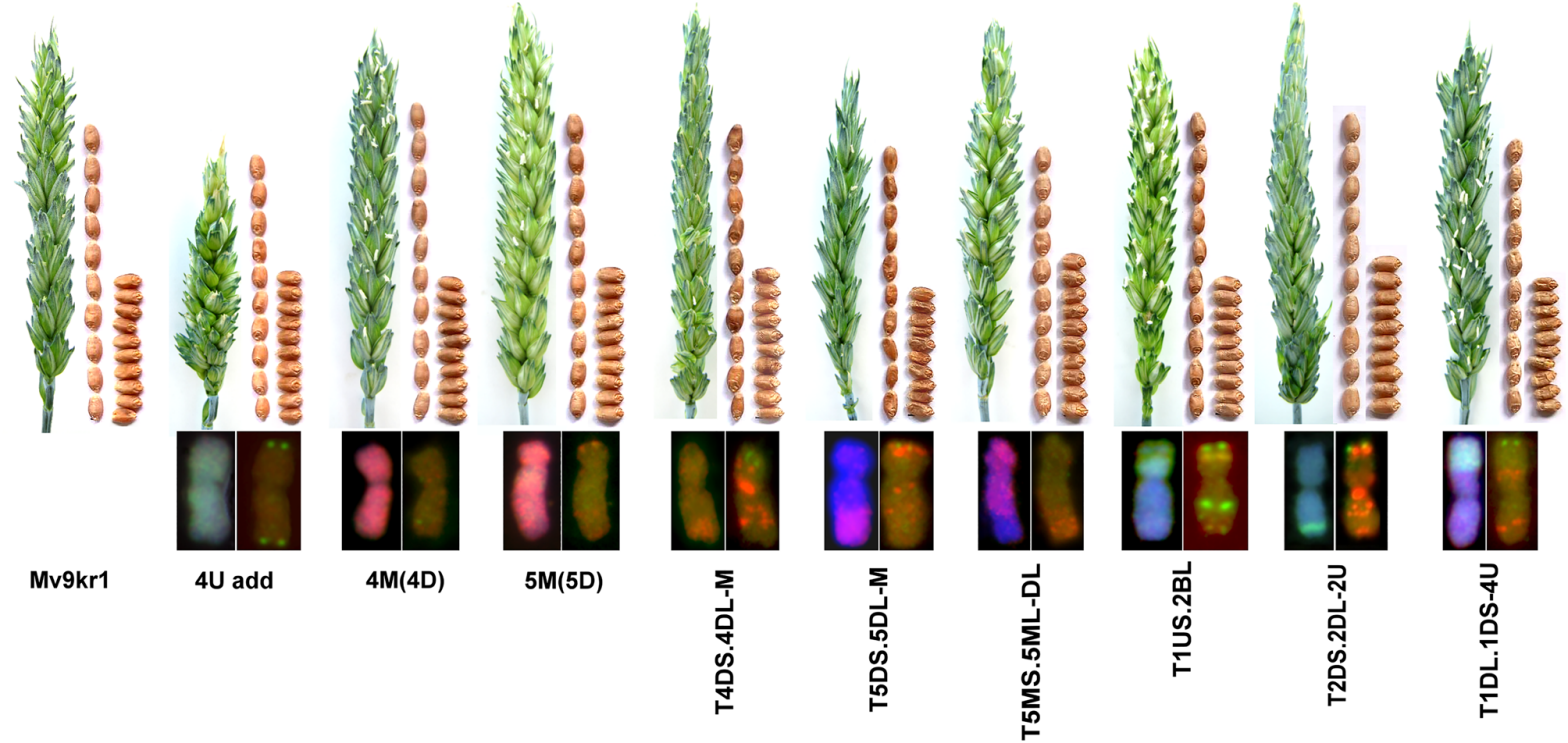
Spike and seed morphology of Mv9kr1, Mv9kr1*-Ae. biuncialis* (MvGB642) 4U addition line (4U), 4M(4D) and 5M(5D) substitution lines, and disomic translocation lines.

Traits related to plant and spike architecture, as well as yield, were investigated under glasshouse conditions (Table 2) for each genotype, and under field condition for addition, substitution, and the 2DS.2DL-2U translocation lines under field condition (Table S1). The spikes of the 4U addition lines have a compact structure but are significantly shorter than the spikes of the wheat parent (Mv9kr1) (Fig. 6, Table 2). These spikes have fewer spikelets than Mv9kr1, resulting in significantly lower grain numbers per spike and per plant. A similar trend was also observed under field conditions. The plant height (Ph) of the 4M(4D) substitution line is significantly shorter than that of the wheat parent under both glasshouse and field conditions. Its spikes are shorter, but only the number of spikelets per main spikes (Spms) differs significantly from wheat. The number of seeds per main spikes (Sepms) and per plant (Sepp), however, is comparable to the Mv9kr1 parent, indicating good fertility. These differences in morphological traits were eliminated under high-input field conditions (Table S1), with the exceptions of plant height and TKW.

**Table 2:** Morphological traits of Mv9kr1, wheat-*Ae. biuncialis* 4U addition (4U), substitutions 4M(4D) and 5M(5D), and translocation lines grown in the glasshouse (2020, Martonvásár).

Genotype	Ph (cm)	T	Lms (cm)	Spms	Sepms	Sepp	TKW (g)
Mv9kr1^#^	58.2 ± 4.7	3.4 ± 0.8	9.6 ± 0.7	23.2 ± 0.7	44 ± 3.2	133.8 ± 22.7	34.4 ± 4.5
4U	50	5	7.5	19	26	81	38.8
4M(4D)^##^	48.8* ± 3.7	4.2 ± 0.7	8.8 ± 1	19.8* ± 1.9	41.2 ± 4.8	130.8 ± 14.7	28.0 ± 3.2
5M(5D)^###^	50.3 ± 3.3	3.7 ± 0.5	10.5 ± 0.8	24.7 ± 0.9	51.7 ± 9.3	134.3 ± 5.8	26.6 ± 5.7
T4DS·4DL-M	51	7	8.5	21	24	66	39.2
T5DS·5DL-M	41	3	9	16	11	30	37
T5MS·5ML-DL	62	6	9.5	20	26	83	37.7
T1US·2BL	47	4	8.5	18	38	150	27.9
T2DS·2DL-2U	48	4	8	17	60	168	25
T1DL·1DS-4U	37	3	9.5	20	5	9	42

^#^Values are the means ± standard deviations of ten measurements.

^##^Values are the means ± standard deviations of five measurements.

^###^Values are the means ± standard deviations of three measurements.

*Significantly different from the value of parental wheat Mv9kr1 at p = 0.05.

The morphological traits of the 5M(5D) substitution line differ from those of the wheat parent in some ways, but due to the small number of samples (3 plants), they did not reach statistical significance (Fig. 6, Table 2). However, in the glasshouse, the substitution line appears to be shorter than the wheat parent, but it is longer in the field. It has compact, awnless, and slightly longer spikes than wheat, with similar or better fertility in terms of number of grains per spike and per plant in both environments.

Two of the Mv9kr1-*Ae. biuncialis* translocation lines (represented by single plants) deserve special attention. The T1US·2BL centric fusion is shorter than the wheat parent. It has shorter spikes with fewer spikelets than wheat, but similar fertility and seed set. T2DS·2DL-2U is also shorter than the wheat parent in both environments. Although its conical spikes have fewer spikelets, its fertility exceeds that of wheat in both glasshouse and field conditions.

Except for the good tillering ability of the T4DS·4DL-M and T5MS·5ML-DL translocations, the other translocation lines yielded less than the wheat parent in terms of yield components. At the same time, their fertility provides enough seeds to maintain these translocations and stabilize the genetic background of wheat through backcrossing. Following the propagation of these lines, the agronomic traits can be studied in details.

## Discussion

Our results confirmed that sequence assemblies of the flow-sorted *Aegilops* chromosomes facilitate marker development to support gene introgression from *Ae. biuncialis* via the production of new wheat-*Aegilops* chromosome addition, substitution, and translocation lines.

### Development of chromosome specific markers

The importance of flow-sorted chromosomes in marker development for mapping alien introgressions was previously demonstrated by Tiwari et al.^46^ who flow sorted 5M^g^S telosomes from wheat-*Ae. geniculata* ditelosomic addition line and identified 2,178 unique SNPs allowing the identification of T5D·5M^g^ translocation. The present work produced gene specific PCR markers suitable to use in laboratories equipped just by a PCR machine and ability for fragment analysis.

Only 7.7% of the 39 EST-based markers tested on the addition lines could be used to select introgression lines, compared to 50% (10 markers) for IT markers and 28% (19 markers) for cDNA-based markers. Our results are in line with previous findings that intron regions of genes have sufficient polymorphism (SNP, InDel) to develop markers suitable for use on various species with similar genomes^47^. COS and PLUG (PCR-based Landmark Unique Gene) markers specific for the intron regions of conserved ortholog genes have previously been used to identify wheat-*Aegilops* introgression lines^31,48,49^. These markers have also been used successfully to identify and trace alien chromosomes or to perform comparative analysis of *Aegilops* species with U and M genomes^32–34^.

The results showed that nearly 40%, 34.5% and 53% of the PCR-tested EST, IT and cDNA markers, respectively, produced amplicons polymorphic between the wheat (Mv9kr1) and *Ae. biuncialis* MvGB642 crossing partners. One reason for the low PCR validation ratio may be sequence variations between wheat (CS) or *Aegilops* (*Ae. umbellulata* AE740/03) genotypes used for in silico primer design and the parental wheat (Mv9kr1) or *Ae. biuncialis* (MvGB642) lines used for the PCR reactions. Another reason for the markers’ reduced validation ratio may be the use of low quality, highly fragmented draft sequence assemblies of *Ae. umbellulata* chromosomes^43^ for marker development. Combining contig assemblies from Illumina short read data with long-read sequencing technologies for scaffolding and data from Hi-C chromatin conformation capture libraries to prepare chromosome pseudomolecules would be a promising pipeline in the future to produce high quality reference assemblies for *Ae. umbellulata* genome, as it was demonstrated recently for *Ae. sharonensis*^40^.

We found that the combined application of molecular markers and genomic in situ hybridization (GISH) is the most optimal approach for selecting wheat genotypes with alien chromosomes in high throughput. The cost of the marker selection could be further reduced by converting PCR markers into Kompetitive Allele Specific PCR (KASP) markers, which do not require fragment analyzing step^50^. Combining GISH with multiple SNP genotyping systems, such as a 35 K SNP chip^51^ and Genotype-by-sequencing (GBS) platforms ^52^, can further increase the resolution and accuracy of the selection process.

The method we used enabled us to identify some translocations, such as the T2DS·2DL-2U or the T1DL·1DS-4U translocations. However, in other cases, the markers used were unable to detect the presence of cytogenetically detectable translocations (e.g., T5DS·5DL-M), implying that the generated markers were not localized on the given M genomic fragment. Additional markers specific to the aforementioned chromosomal regions will need to be developed.

### Development of new wheat-*Ae. biuncialis* genetic materials

M-genome chromosomes were more abundant in the BC_3_F_3_ population than the U-genome chromosomes. This phenomenon may be related to the wheat-*Aegilops* cross-genome homology, which is higher in the case of the M genome than those of the U genome, which contains multiple re-arrangements relative to D genome of wheat^32,34,43,53^. The predominant pairing affinity of wheat chromosomes with M-genome chromosomes relative to U-genome chromosomes at meiotic metaphase I of wheat × *Ae. biuncialis* F_1_ hybrids ^54^ also indicated a closer wheat-M macrosyntenic relationship. During the crossing programs, a lower frequency of wheat-U chromosome associations resulted in abundant univalent formation of U chromosomes, which can be eliminated with higher probability from the offsprings.

The present study also indicated that chromosomes 4M and 5M were dominantly present in the population. The preferred inheritance of some alien chromosomes, which is common in wheat × *Aegilops* hybrid progenies, is frequently associated with the effect of gametocidal genes present in the given chromosome. Gametocidal genes ensure their existence in the host by causing selective abortion of gametes that do not carry them; as a result, they are preferentially transmitted to the offspring^55–58^. Gametocidal genes were detected on several chromosomes of different *Aegilops* species, including chromosome 4M of *Ae. geniculata,* another allotetraploid species with U and M genomes* (*U^g^U^g^M^g^M^g^)^59–62^ indicating that chromosome 4M of *Ae. biuncialis* may have a similar effect. The gametocide effect of chromosome 4M was also supported by the frequent observation of aberrant chromosomes resulted from random chromosome breakages.

From breeding point of view, gametocidal genes have received special attention as several cytogenetic materials have been developed using the gametocidal system, including a set of deletion lines for hexaploid wheat^56^ and wheat-alien introgression lines^63–65^. Kwiatek et al.^62^ produced wheat-rye translocations using triticale lines containing the 4M^g^ chromosome of the *Ae. geniculata* in the monosomal state. However, the confirmation of the presence of the gametocidal gene in our wheat-*Ae. biuncialis* 4M^b^ and/or 5M^b^ addition lines needs further evidences.

The present work developed new hexaploid wheat-*Ae. biuncialis* 4U disomic addition, as well as 4M(4D) and 5M(5D) disomic substitution lines. We also produced six wheat-*Ae. biuncialis* chromosome translocations (T4DS·4DL-M, T5DS·5DL-M, T5MS·5ML-DL, T1US·2BL, T2DS·2DL-2U, T1DL·1DS-4U), which occurred in disomic form in separate lines. These cytogenetic stocks significantly contribute to increase wheat genetic variability.

The significant macro collinearity of 4M and 5M chromosomes with the chromosomes of wheat homeologous groups 4 and 5, respectively^43^, implies that gene composition and order of these *Aegilops* and wheat chromosomes are similar. In line with this, the similar spike structure and fertility of the 4M(4D) and 5M(5D) substitution lines to the wheat parent also indicate that the 4M and 5M chromosomes compensate well for the loss of genes localized on the 4D and 5D chromosomes, respectively. In this respect, several QTLs have been mapped to wheat homeologous groups 4 and 5 chromosomes, which play a key role in crop yield formation ^66^, environmental adaptation^67–69^ and abiotic stress tolerance^70^. QTLs associated with yield components, including tillering ability and grain number per spike have been mapped on 4D and 5D chromosomes^71,72^ and on chromosomes 4A, 5B, and 5D^73–75^, respectively.

Plant height also has an impact on wheat yield, and exploiting the role of dwarfing or reduced height (*Rht*) loci resulted in one of the most significant breakthroughs in modern plant breeding during the Green Revolution^76^ of the twentieth century. The *Rht1* and *Rht2,* are two of the most commonly used dwarfing genes in wheat, have been located on the 4BS and 4DS chromosome arms, respectively^77,78^. In the present study, replacement of the chromosome 4D with 4M caused a 10 cm reduction in plant height in the substitution line, which may be attributed to the effect of a putative *Aegilops Rht* gene variant present on the 4M chromosome.

The regulation of flowering time is one of the crucial elements in wheat's adaptation to various ecogeographical environments^79,80^. The most essential factors in determining flowering time in cereals are the *Vrn* genes that regulate the need for vernalization (the need for the cold period that triggers the transition from the vegetative to the generative phase). The major *Vrn* genes in wheat are *Vrn-A1*, *Vrn-B1*, and *Vrn-D1*, which are located on the long arms of chromosomes 5A, 5B, and 5D, respectively^67,81,82^.

Our previous research on wheat-*Ae. geniculata* addition lines suggests that chromosomes 5U^g^ and 5M^g^ have a positive effect on the grain dietary fiber composition and β-glucan content of hexaploid wheat^17^, implying that Mv9kr1-*Ae. biuncialis* 5M^b^(5D) substitution line could also be useful gene source for the production of functional foods.

These examples indicates that new allelic variants of agriculturally important QTLs and genes can be introduced into wheat by *ph*-induced homoeologous recombination of chromosomes 4D and 5D with the collinear chromosomes 4M and 5M, respectively. The future use of *ph1b* mutant in wheat Mv9kr1 line may allow for the production of 4D/4M and 5D/5M recombinations without changing the wheat genetic background^54^.

## Conclusion

The sequences of flow sorted chromosomes allowed the development of a significant amount of gene-based markers specific for the U and M genome chromosomes of *Aegilops* species. Using markers PCR-validated on the parental genotypes and wheat-*Aegilops* chromosome addition lines, we selected new wheat-*Ae. biuncialis* disomic 4U addition, 4M(4D), and 5M(5D) substitutions, as well as several introgression lines. The morphology and fertility of the spikes indicated that the *Aegilops* 4M or 5M chromosomes compensated well for the loss of wheat chromosomes 4D and 5D, respectively. The new cytogenetic stocks may serve as a suitable genetic resource to introgress wild alleles of key genes determining some important agronomic traits into wheat through chromosome engineering.

## Methods

### Plant material

*Ae. umbellulata* (2*n* = 2*x* = 14, UU) accession AE740/03 provided by the Institute of Plant Genetics and Crop Plant Research (Gatersleben, Germany) and *Ae. comosa* (2*n* = 2*x* = 14, MM) accession MvGB1039 maintained in the Martonvásár Cereal Gene Bank (Martonvásár, Hungary) were used for chromosome sorting, sequencing and marker design.

The chromosomal positions of the markers were validated by PCR on the following wheat-*Aegilops* chromosome addition lines: CS-*Ae. umbellulata* 1U, 2U, 4U, 5U, 6U, 7U (kindly provided by Dr. Steve Reader (John Innes Centre, Norwich, UK).); Mv9kr1-*Ae. biuncialis* MvGB642 3U^22^ and CS-*Ae. geniculata* 1M–7M addition lines ^57^ were kindly provided by Dr. Bernd Friebe (Kansas State University, Manhattan, Kansas, USA).

The *Ae. biuncialis* (2*n* = 4*x* = 28, U^b^U^b^M^b^M^b^) accession MvGB642 was collected from an area 1160 m above the sea level near Slinfah, Syria.

The winter wheat (*T. aestivum*) line Mv9kr1 carrying a recessive crossability allele *kr1 *^83^ was crossed with *Ae. biuncialis* MvGB642 to develop a BC_3_F_3_ population (Fig. S2). The BC_3_F_3_ progeny plants were characterized by GISH, FISH, and molecular markers.

### In situ hybridization

Mitotic chromosome preparations were made from the root tips of BC_3_F_3_ plants according to Molnár et al. ^42^. Genomic DNA was isolated using the Quick Gene-Mini80 system (FujiFilm, Osaka, Japan) according to the manufacturer’s instructions. Total genomic DNA from *Ae. umbellulata* (2*n* = 2*x* = 14; UU) was labeled with biotin (BioPrime™ DNA Labeling System, Invitrogen™, Carlsbad, USA) and used as the U-genomic probe. Total genomic DNA from *Ae. comosa* (2*n* = 2*x* = 14; MM) was labeled with digoxigenin-11-dUTP (Roche) using the Random Primed DNA Labeling Kit (Roche) following the manufacturer’s instructions, and used as the M-genomic probe. The repetitive DNA sequences of Afa-family and pSc119.2 were amplified using PCR and labelled with digoxigenin-16-dUTP (Roche) and biotin-11-dUTP (Roche), respectively ^84,85^. The 18S-5.8S-26S rDNA clone pTa71 was labeled in equal proportions with biotin-11-dUTP and digoxigenin-16-dUTP by nick translation ^86^. Digoxigenin and biotin were detected using anti-digoxigenin-rhodamine Fab fragments (Roche) and streptavidin–FITC (Roche), respectively.

Sequential GISH and FISH on BC_3_F_3_ lines were performed as described by Molnár et al. ^26^. Briefly, the hybridization solution (25 μl per slide) for GISH experiments contained 70 ng of each U- and M-genomic labeled probe together with unlabeled wheat genomic DNA to block unspecific hybridization at a concentration of 50× that of the probe. After the hybridization (42 °C, overnight) and post treatments, the chromosomes were counterstained with 2 μg/ml DAPI (4’-6-diamino-2-phenylindole) and mounted in antifade solution. Following GISH, FISH was performed using probes for DNA repeats Afa-family, pSc119.2, and pTa71. Fluorescence signals were investigated by an AxioImager M2 epifluorescence microscope (Zeiss, Oberkochen, Germany) equipped with filter sets for detecting DAPI, FITC, and Rhodamine signals. Images were captured with a Zeiss AxioCam MRm CCD camera and processed with Zeiss AxioVision 4.8.2. software (Zeiss).

### Flow cytometric chromosome sorting, sequencing and marker design

Flow cytometric sorting of mitotic metaphase chromosomes from *Ae. comosa* MvGB1039 and *Ae. umbellulata* AE740/03 was carried out as previously described by Molnár et al. ^34^ and Said et al. ^87^. The chromosomal DNA was purified according to Šimková et al. ^88^, sequenced on an Illumina NovaSeq 6000 (*Ae. comosa*) or HiSeq2000 (*Ae. umbellulata*), and assembled as described by Said et al. ^43^. In the present study, assembled chromosome contigs of *Ae. umbellulata* AE740/03 (https://doi.org/10.5061/dryad.70rxwdbwc) and *Ae. comosa* MvGB1039 (https://doi.org/10.5061/dryad.wpzgmsbk9) were used to design molecular markers.

Three strategies were used to design PCR markers specific for the U- and M-chromosomes of *Aegilops*. For the first strategy (1) ESTs specific for the D genome chromosomes of hexaploid wheat were selected from publicly available database (https://wheat.pw.usda.gov/cgi-bin/westsql/map_locus.cgi). A total of 4514 D-genome specific wheat EST sequences (1D: 633; 2D: 742; 3D: 719; 4D: 586; 5D: 622; 6D: 485; 7D: 727) were aligned to the genomic sequences of the *Ae. umbellulata* AE740/03 (Data S1) using BLASTn with the program Ragged Genes Genome Explorer (version 2.2.24) which is a graphic interface for NCBI’s BLAST command line tool ^89^. BLASTn hits that met certain criteria (InDel polymorphism ≥ 5 bp and wheat-*Aegilops* homology ≥ 70%) were selected, and identification of InDels to design primers to the polymorphic regions was carried out using pairwise alignments of the selected ESTs and *Aegilops* contigs using UGENE (v.1.27.0) and Primer3. The selected ESTs polymorphic between wheat and *Aegilops* together with their PCR primers were summarized in Data S2.

The second marker development strategy (2) was based on the polymorphism between the intron regions of the *Ae. umbellulata* genes and the homologous wheat genes (Intron Targeting markers: IT markers). During marker development, we used annotated gene models of *Ae. umbellulata*. For automated primer design, bedtools (v.2.26.0), primer3 (v.2.3.6) and a custom R script with the package devtools were used. In the first step, BLASTn was used to search wheat homologues of the *Ae. umbellulata* genes (*Aegilops* genes were used as query sequences for BLASTn searches in the wheat reference genome sequence [RefSeq v1.0^37^], and then the positions of the introns were determined). The polymorphic 600 bp region of genes that contains the last 100 bp of the first exon and the first 500 bp of the first intron (> 500 bp) was selected and saved in a multifasta file. Primer pairs were generated for these sequences. The forward primer was located on the first 100 bp (exon-specific) region and the reverse primer was located on the subsequent 500 bp (intron-specific) region. A total of 84 primer pairs (12 markers per chromosome) were randomly selected. The oligonucleotide sequences were synthesized by Integrated DNA Technologies (Coralville, Iowa, USA).

The third marker development strategy was based on a polymorphism between cDNA sequences of wheat genes and *Ae. umbellulata* and *Ae. comosa* sequences. In a previous study, Said et al.^43^ mapped forty-three ortholog genes on M- and U-genome progenitors, *Ae. comosa* and *Ae. umbellulata,* respectively, using single-gene FISH with fluorescently labeled cDNA sequences as probes. In order to validate the cytogenetic maps, PCR markers were developed for the cytogenetically mapped genes. From this set of markers, 19 were selected and used to identify or confirm the cytological results in the BC_3_F_3_ plants.

The synthesized primer pairs for both EST-based markers and IT markers were tested by PCR on the parental genotypes Mv9kr1, *Ae. biuncialis* MvGB642, and *Ae. umbellulata* AE740/03. The primer pairs that produced *Aegilops*-specific amplicons were tested further by PCR on wheat *Aegilops* addition lines (CS-*Ae. umbellulata* 1U, 2U, 4U, 5U, 6U, 7U, Mv9kr1-*Ae. biuncialis* MvGB642 3U, and CS-*Ae. geniculata* 1M–7M addition lines) representing complete sets of U- and M-genome chromosomes in wheat genetic background, and on the Mv9kr1-*Ae. biuncialis* MvGB642 amphiploid. The reaction mixture (15 μl) containing 0.2 μmol/l of forward and reverse primers, as well as the thermal reaction profile used for the PCR reactions, were described by Molnár et al. ^90^. PCR amplicons were detected along with a 75–400 bp Range DNA Ladder using a Fragment Analyzer Automated CE System (Advanced Analytical Technologies, Ames, USA) and analyzed with PROsize v2.0 software (Advanced Analytical Technologies).

### Statistical analysis

The wheat (Mv9kr1)-*Ae. biuncialis* MvGB642 addition, substitution and translocation lines developed by the present study were grown in glasshouse as described by Rakszegi et al. ^17^.

The wheat (Mv9kr1)-*Ae. biuncialis* MvGB642 4U addition, 4M(4D) and 5M(5D) substitution, and 2DS·2DL-2U translocation lines were also grown in a high-input field nursery (Bulgárföld, Martonvásár, Hungary, geographic coordinates: 47°19′39′′ N, 18°47′01′′ E) in the 2022–2023 season. In the field experiment, 250 grains of each genotype were sown in 2 m^2^ plots (5 × 2 m rows; 50 grains per row, row distance: 0.15 m). The trial plots were machine-planted (HEGE-80 plot driller) and hand-harvested. The clayey chernozem texture soil (pH  7.25) contained 2.8% w/w humus, 210 mg/kg P_2_O_5_ and 210 mg/kg K_2_O.

The results for the glasshouse experiments are the means ± standard deviation of three [5M(5D)], five [4M(4D)] and ten (Mv9kr1) measurements per genotype for plant height (Ph), tillering (T), length of main spike (Lms), spikelets/main spike (Spms), seeds/main spike (Sepms), seeds/plant (Sepp) and thousand kernel weight (TKW). Differences between the substitution lines and the wheat parental line Mv9kr1 were determined by means of Student’s t test for paired data at the P = 0.05 and P = 0.01 significance levels. In the case of field experiments, the results are the means ± standard deviations of ten plants per genotypes. The significant differences between the genotypes were calculated using the Tukey’s post hoc test of the IBM SPSS Statistics 20.0 software (SPSS Inc., Chicago, IL, USA).

### Supplementary Information

Supplementary Information 1.

Supplementary Information 2.

Supplementary Information 3.

Supplementary Information 4.

Supplementary Information 5.

## Data Availability

All data is contained within the present article and the supplementary material.
