# Supplementary material for: Chromosome genomics facilitates the marker development and selection of wheat-Aegilops biuncialis addition, substitution and translocation lines
Source: Sci Rep. 2023 Nov 22;13:20499. doi: 10.1038/s41598-023-47845-8 (PMC10665447; doi:10.1038/s41598-023-47845-8)
Supplement: Supplementary file 5 — Supplementary Information 5. [file 41598_2023_47845_MOESM5_ESM.docx]

**Supplementary Figures and Table**

**Chromosome genomics facilitates the marker development and selection of wheat-*Aegilops biuncialis* addition, substitution and translocation lines**

András Farkas^1†^, Eszter Gaál^1†*^ ,László Ivanizs^1^, Nicolas Blavet^2^, Mahmoud Said^2,3^, Kateřina Holušová^2^, Kitti Szőke-Pázsi^1^, Tamás Spitkó^1^, Edina Türkösi^1^, Klaudia Kruppa^1^, Péter Kovács^1^, Éva Darkó^1^, Éva Szakács^1^, Jan Bartoš^2^, Jaroslav Doležel^2^, István Molnár^1,2^

^1^Department of Biological Resources, Centre for Agricultural Research, Eötvös Lóránd Research Network, Martonvásár 2462, Hungary

^2^Institute for Experimental Botany, Centre of the Region Haná for Biotechnological and Agricultural Research, Olomouc 779 00, Czech Republic

^3^Field Crops Research Institute, Agricultural Research Centre, 9 Gamma street, Giza, 12619 Cairo, Egypt

^*^corresponding author: [gaal.eszter@atk.hu](mailto:molnar.istvan@atk.hu)

^†^These authors contributed equally to this work

**Figure S1** PCR validation of gene-based (EST, IT, cDNA) markers developed by the use of *Ae. umbellulata* and *Ae. comosa* chromosomal sequences. Representative digital capillary electrophoretic patterns shows the PCR amplicons produced by the IT markers on the crossing partner hexaploid wheat line Mv9kr1 and *Ae. biuncialis* (MMUU) MvGB642 accession (A). Markers polymorphic between wheat and *Ae. biuncialis* were further tested on wheat (CS)-*Ae. umbellulata* and wheat (CS)-*Ae. geniculata* addition lines representing the whole set of M- or U-genome chromosomes together with parental wheat Chinese Spring (CS) and the Mv9kr1-*Ae. biuncialis* amphiploid (Amph) genotypes (B). Finally, *Aegilops*-specific markers with validated chromosomal locations were used for genotyping of Mv9kr1-*Ae. biuncialis* MvGB642 BC_3_F_3_ population (201008-201201) together with their parental wheat (Mv) and Aegilops (Ae) lines and their amphiploid (Amp) genotype. A 35-500 bp DNA ladder was used as a molecular-weight size marker to estimate the fragment size.


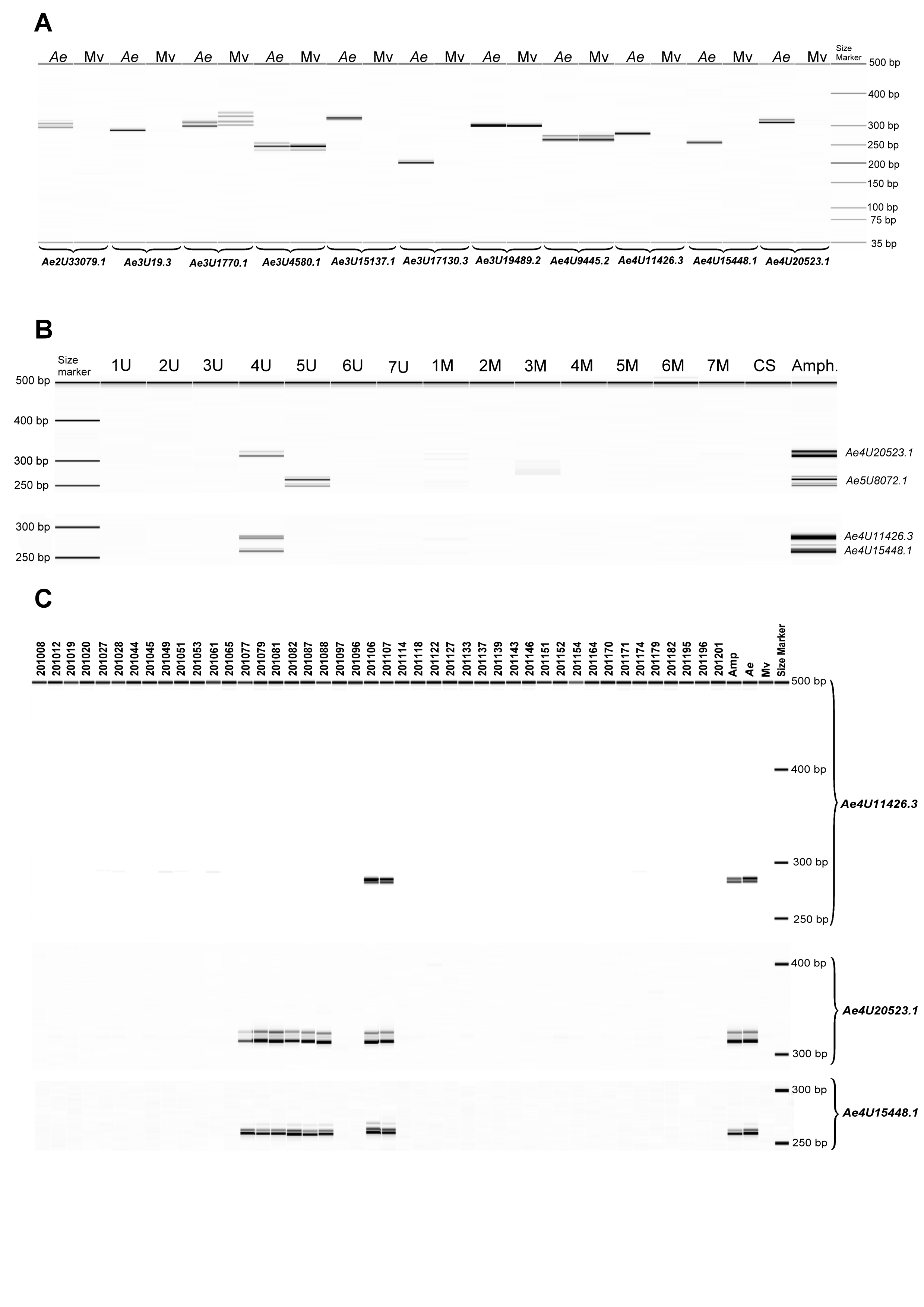


**Figure S2** Flow diagram of the development of Mv9kr1-*Ae. biuncialis* MvGB642 BC_3_F_3_ population.


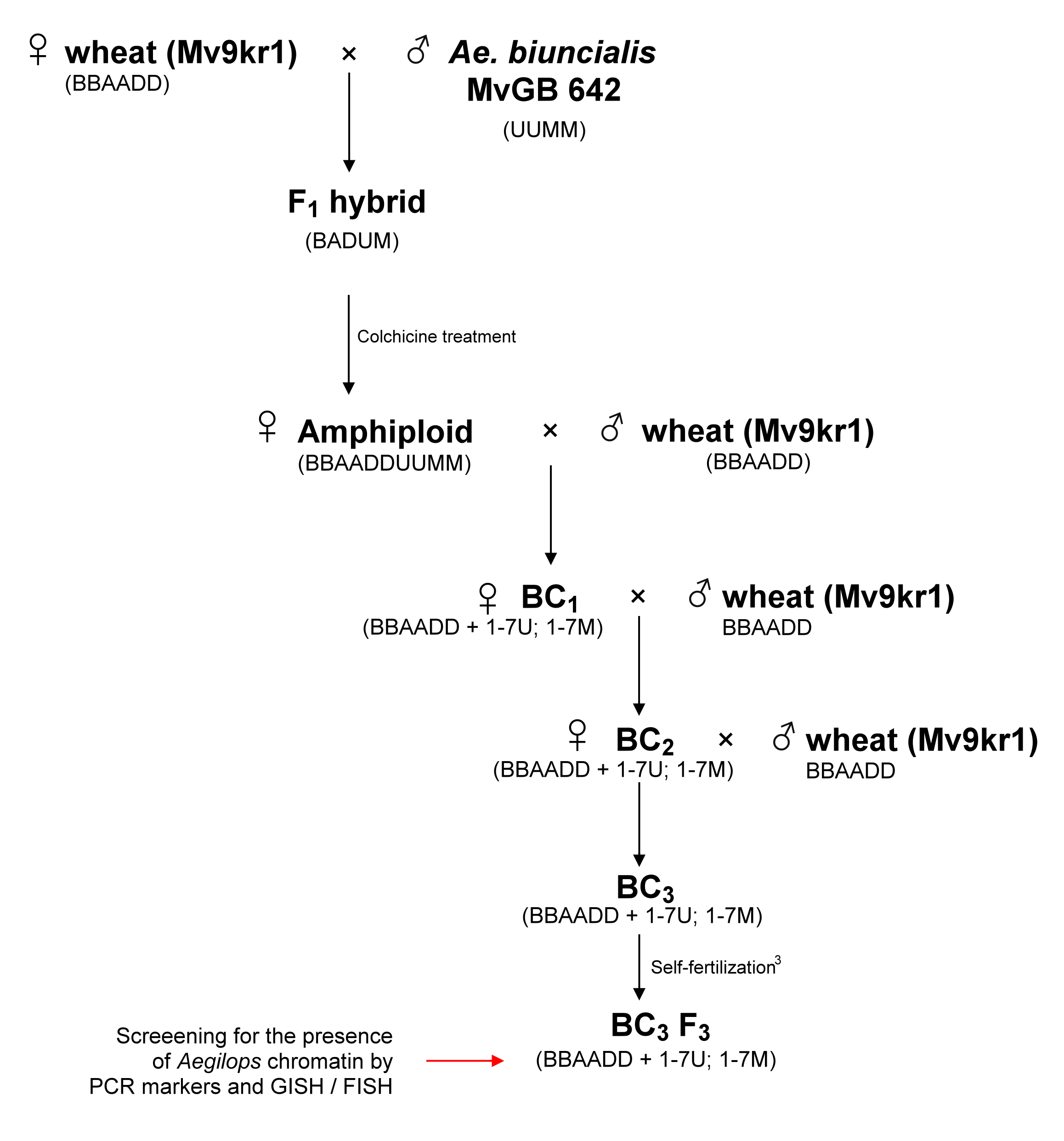


**Figure S3** FISH (A) and GISH (B) patterns on mitotic chromosomes of the Mv9kr1-*Ae. biuncialis* MvGB642 BC_3_F_3_ genotype 201049. The line carries a monosomic 5MS.5ML-2DL translocation, a wheat-M centric fusion (RobM), and a monosomic 4M chromosome. FISH was performed with DNA repeat probes for Afa-family (red), pSc119.2 (green), and pTa71 (yellow). In the case of GISH, total genomic DNA of *Ae. umbellulata* and *Ae. comosa* was labeled and visualized with green and red fluorescence, respectively, while unlabelled and DAPI stained wheat chromosomes were visualized in blue. Scale bar =10 μm.


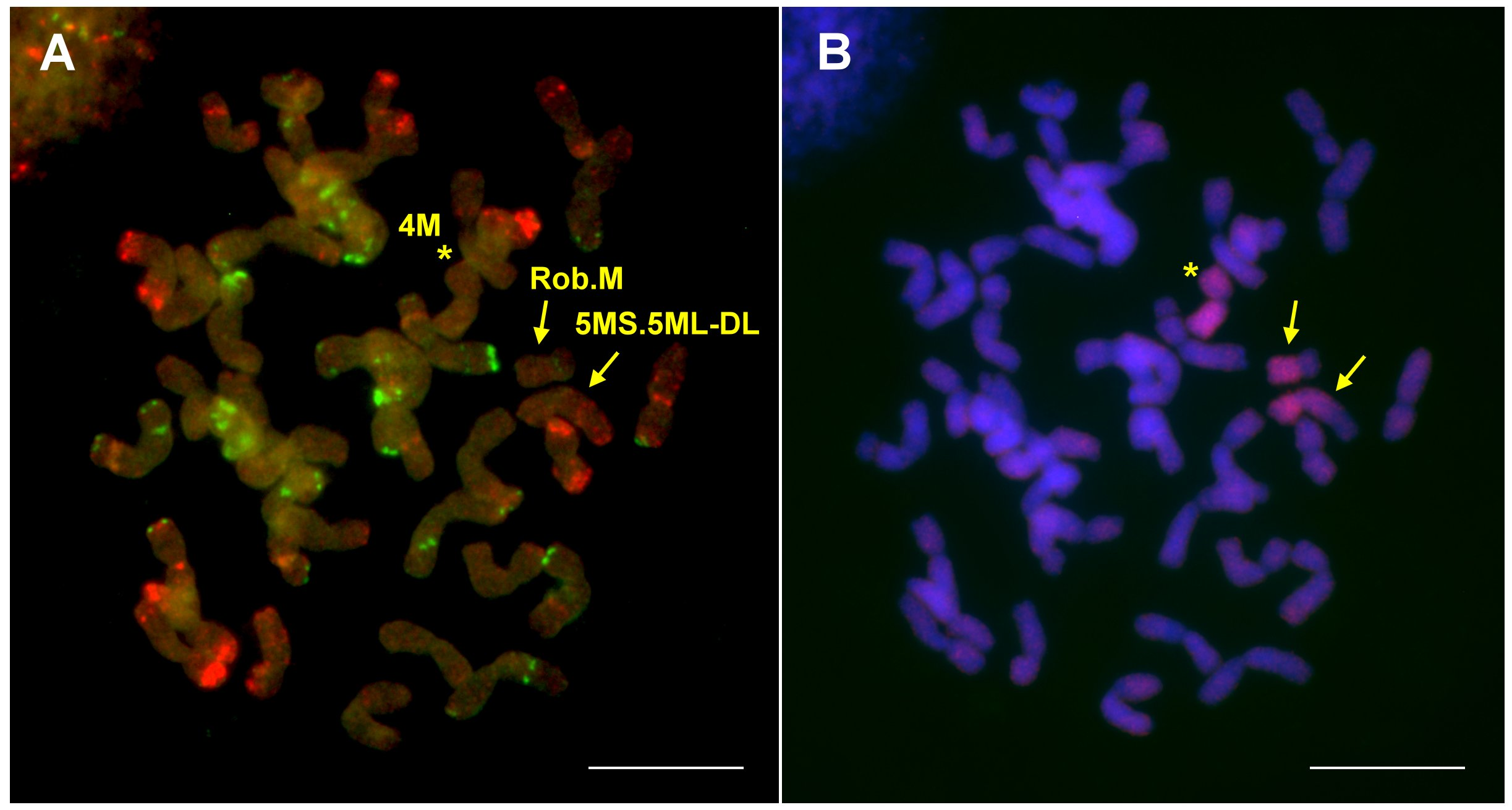


**Table S1** Morphological traits of Mv9kr1, wheat-*Ae. biuncialis* 4U addition (4U), substitutions 4M(4D) and 5M(5D), and translocation lines grown in field nursery (2023, Martonvásár).

| **Genotype** | **Plant height (cm)** | **Number of spikes/plant** | **Length of main spike (cm)** | **Number of Spikelets/main spike** | **Number of Seeds/main spike** | **Number of Seeds/plant** | **Fertility (seeds/spikelet)** | **TKW (g)** |
| --- | --- | --- | --- | --- | --- | --- | --- | --- |
| **Mv9kr1** | 95.1  ±5.90 b | 13.6  ±2.37 a | 11.0  ±1.00 b | 21.8  ±2.15 a | 50.4  ±5.48 a,b | 400.0  ±86.11 a | 2.3  ±0.33 b | 31.1  ±1.98 a |
| **4U** | 59.4  ±5.68 d | 5.7  ±1.16 c | 9.5  ±0.69 c | 21.9  ±1.37 a | 46.1  ±19.89 b | 140.3  ±53.69 b | 2.1  ±0.82 b | 10.6  ±6.88 c |
| **4M(4D)** | 81.3  ±5.08 c | 13.7  ±3.59 a | 10.8  ±0.62 b | 21.2  ±1.13 a | 47.0  ±6.80 b | 420.1  ±121.17 a | 2.2  ±0.22 b | 20.8  ±14.1 b |
| **5M(5D)** | 103.4  ±4.14 a | 11.4  ±4.72 a,b | 12.1  ±0.60 a | 21.9  ±0.88 a | 58.0  ±3.65 a,b | 505.5  ±184.54 a | 2.6  ±0.13 a | 27.0  ±3.13a.b |
| **T2DS∙2DL-2U** | 84.7  ±2.63 c | 8.1  ±3.28 b,c | 9.8  ±0.63 c | 20.6  ±1.07 a | 60.9  ±10.02 a | 356.1  ±165.32 a | 3.0  ±0.40 a | 31.4  ±1.34 a |

Values are the means ± standard deviations of 10 measurements. Values with the same letters in a column do not differ significantly by Tukey’s post hoc test (α = 0.05).
